# Predator metamorphosis and its consequence for prey risk assessment

**DOI:** 10.1093/beheco/arae014

**Published:** 2024-03-06

**Authors:** Himal Thapa, Adam L Crane, Gabrielle H Achtymichuk, Sultan M M Sadat, Douglas P Chivers, Maud C O Ferrari

**Affiliations:** Department of Biology, University of Saskatchewan, Science Pl., Saskatoon, SK S7N 5C8, Saskatchewan, Canada; Department of Veterinary Biomedical Sciences, Western College of Veterinary Medicine, University of Saskatchewan, 52 Campus Dr, Saskatoon, SK S7N 5B4, Saskatchewan, Canada; Department of Veterinary Biomedical Sciences, Western College of Veterinary Medicine, University of Saskatchewan, 52 Campus Dr, Saskatoon, SK S7N 5B4, Saskatchewan, Canada; Department of Biology, University of Saskatchewan, Science Pl., Saskatoon, SK S7N 5C8, Saskatchewan, Canada; Department of Biology, University of Saskatchewan, Science Pl., Saskatoon, SK S7N 5C8, Saskatchewan, Canada; Department of Veterinary Biomedical Sciences, Western College of Veterinary Medicine, University of Saskatchewan, 52 Campus Dr, Saskatoon, SK S7N 5B4, Saskatchewan, Canada

**Keywords:** alarm cues, generalization, predaceous diving beetle, predator odor, tiger salamander, wood frog tadpole

## Abstract

Living with a diverse array of predators provides a significant challenge for prey to learn and retain information about each predator they encounter. Consequently, some prey respond to novel predators because they have previous experience with a perceptually similar predator species, a phenomenon known as generalization of predator recognition. However, it remains unknown whether prey can generalize learned responses across ontogenetic stages of predators. Using wood frog tadpole (*Lithobates sylvaticus*) prey, we conducted two experiments to explore the extent of predator generalization of different life stages of two different predators: (1) predacious diving beetles (*Dytiscus* sp.) and (2) tiger salamanders (*Ambystoma mavortium*). In both experiments, we used chemical alarm cues (i.e., injured conspecific cues) to condition tadpoles to recognize the odor of either the larval or adult stage of the predator as risky. One day later, we tested tadpoles with either the larval or adult predator odor to determine whether they generalized their learned responses to the other life stages of the predator. Tadpoles generalized between larval and adult beetle odors but failed to generalize between larval and adult salamander odors. These results suggest that the odor of some predator species changes during metamorphosis to an extent that reduces their recognisability by prey. This “predator identity reset” increases the number of threats to which prey need to attend.

## Teaser

Wood frog tadpoles can generalize learned antipredator responses across the life stages of some predator species but not all. When individuals were conditioned to recognize the odor of larval or adult predacious diving beetles as risky, they subsequently showed antipredator response to the odor of both life stages. However, tadpoles did not generalize their learned responses across the life stages of the tiger salamander.

## INTRODUCTION

Many prey species have evolved phenotypically plastic antipredator responses to aid survival when exposed to predators. Some develop morphological defenses (Harvell 1984; Fässler and Kaiser 2008) or alter the timing of their life history transitions to avoid predators (Chivers and Smith 1998; Kelley and Magurran 2003; Bierbach et al. 2011). Others employ antipredator behaviors to discourage or evade predator attacks (Lima and Dill 1990; Ferrari et al. 2009). Regardless of how prey respond to predation risk, such responses depend first on the prey’s ability to correctly detect and recognize the threat (Endler 1991). Understanding how prey perceive and then react to predation threats has gained substantial interest from ecologists through both evolutionary and mechanistic lenses (Mitchell et al. 2017; Carthey and Blumstein 2018).

In some instances, prey recognize predators without having a previous encounter, indicating innate predator recognition (Veen et al. 2000; Gall and Mathis 2010; Gerlai 2010). Innate predator recognition is thought to occur in less disturbed habitats with a constant predator community, and thus, predators and prey have a shared evolutionary history of co-existence (Kats and Ferrer 2003; Wisenden 2003). In contrast, when prey fail to recognize predators innately, they need to learn cues from the predator to recognize it correctly (Blumstein et al. 2004; Brown and Chivers 2005; Cornell et al. 2011; Crane and Ferrari 2013). Prey have many ways to label novel predators as threatening. Surviving a single predator attack or sensing visual or chemical cues from an attack on another individual is often enough for prey to learn predator recognition (Crane and Ferrari 2013). Chemical cues released from injured prey, commonly known as “alarm cues,” can also facilitate predator recognition learning (Ferrari et al. 2010a; Roberts and de Leaniz 2011). These cues are a suite of chemicals produced and sequestered in the tissues (often the epidermis) of many prey species and are released upon damage from a predator attack, thus serving as a reliable indicator of a predator risk (Ferrari et al. 2010b). When alarm cues are detected by nearby conspecifics, they can elicit immediate and robust antipredator behaviors, which increase their probability of surviving the predator encounter (Chivers and Smith 1998; Mirza and Chivers 2000; Greggor et al. 2019). These cues are also released in the excreta of predators, often referred to as “diet cues” (Mathis and Smith 1993; Chivers et al. 1996). Prey can learn to identify a predator odor as risky when it is paired with alarm cues (Wisenden 2003). This learning process has received considerable attention in aquatic systems (Ferrari et al. 2010b).

Prey living in diverse communities are exposed to a number of potential threats and must quickly learn to identify predators. One cognitive rule that prey can use to facilitate this task is to make an “educated guess” whether a novel species is likely to be threatening by evaluating the similarity of the cues between this new species and a known predator (Griffin et al. 2001; Ghirlanda and Enquist 2003; Ferrari et al. 2007b). This concept, known as “generalization of predator recognition,” has been demonstrated using chemical and visual predator cues in a variety of taxa, including mammals (Griffin et al. 2001; Saxon-Mills et al. 2018), fishes (Chivers and Smith 1998; Ferrari et al. 2007a; Mitchell et al. 2015), amphibians (Ferrari and Chivers 2009a; Davis et al. 2012), and reptiles (Webb et al. 2009). For example, predator naïve tammar wallabies (*Macropus eugenii*) learned to recognize a red fox (*Vulpes vulpes*) as a predator and were later found to respond to similar-looking feral cats (*Felis catus*) but not juvenile goats (*Capra hircus*) (Griffin et al. 2001). Likewise, Ferrari et al. (2007a) found that predator-naïve fathead minnows (*Pimephales promelas*) that were previously conditioned to recognize lake trout (*Salvelinus namaycush*) as risky, subsequently displayed an antipredator response to brook trout (*Salvelinus fontinalis*) and rainbow trout (*Oncorhynchus mykiss*) unlike their predator-naïve counterparts.

Predators with complex life histories may pose a major challenge to predator recognition by prey because ontogenetic changes may make previously learned information irrelevant or inaccurate. These life-history transitions are often coupled with changes in habitat type and spatio-temporal niche, which can also result in a diet switch (Islam and Tanaka 2006). Most anurans, for example, are planktivorous or herbivorous during their larval stage, becoming carnivorous after metamorphosis (Alford 1999), whereas salamanders are strictly carnivorous throughout their life (Brophy 1980; Dolmen and Koksvik 1983). Moreover, different ontogenetic stages of a predator may pose a differential threat to a shared prey (Aditya and Saha 2006; Bofill and Yee 2019). For instance, adult predaceous diving beetles (*Laccophilus fasciatus*) consume late-instar mosquito larvae at a higher rate when compared with their larval form (Bofill and Yee 2019). From a prey’s point of view, maintaining predator recognition despite the changes that predators undergo is important, as learning is costly, and having to re-learn a threat makes prey vulnerable to predation risk (Brown and Chivers 2005).

Some of the most spectacular life-history changes occur in amphibians and holometabolous insects (i.e., those undergoing “true” metamorphosis). Metamorphosis is characterized as a rapid switch in the ontogeny of an individual, coupled with changes in morphology, physiology, and behavior (Wilbur 1980). For many species, the entire body is rebuilt (Rolff et al. 2019). For example, the aquatic larvae of predacious diving beetles (Family Dytiscidae) live underwater, respiring through the cuticle and tracheal gills before undergoing a drastic body re-structuring at metamorphosis (Korschelt 1924). The adult stage can leave the water and fly but continues to forage in aquatic environments by diving into the water while holding an air bubble connected to a sub-elytral air chamber for respiration (White 2009). Similarly, metamorphosis in amphibians usually allows an aquatic organism to become terrestrial (Gilbert 2000). During metamorphosis, salamanders may undergo changes such as tail fin resorption, the loss of external gills or gill slits, and the development of lungs (Weber 1967; Norman 1985; Rosenkilde 1985; Kaltenbach 1996; Vitt and Caldwell 2014; Parsley and Goldberg 2023). In pond-breeding salamanders (Family: Ambystomatidae), the larval stage inhabits the aquatic environment and begins living terrestrially after metamorphosis, although they return to the aquatic environment for reproduction (Vitt and Caldwell 2014). However, to our knowledge, no study has reported whether prey can maintain learned recognition of a predator that undergoes metamorphosis.

Here, we report the results of two experiments testing whether predator metamorphosis affects recognition by wood frog tadpoles (*Lithobates sylvaticus*), a model prey species in research on predator-recognition learning (e.g., (Ferrari et al. 2016; Crane et al. 2017)). For predators, we used the predaceous diving beetle (*Dytiscus* sp.) in experiment 1, and the tiger salamander (*Ambystoma mavortium*) in experiment 2. Both species forage on tadpoles during their pre- and post-metamorphic life stages (Yee 2010; Bofill 2014). However, previous studies from our lab have reported that wood frog tadpoles did not innately recognize either tiger salamander or beetles as risky, and thus recognition of these predators must be learned (Ferrari et al. 2008b; Crane et al. 2017). For the present study, tadpoles were first conditioned with alarm cues to recognize the odor of either the larval or adult stage of the predator, or they underwent a sham conditioning (alarm cues paired with control water). The following day, tadpoles were tested for their behavioral responses to the larval or adult odor (i.e., a 3 × 2 design). Based on the studies mentioned above, we hypothesized that the odor profile of the predaceous diving beetle and the tiger salamander would not vary much between ontogenetic stages. As a result, we predicted that tadpoles would generalize their learned antipredator response across the different predator life stages.

## MATERIALS AND METHODS

### Test species and maintenance

We collected predator-naïve tadpoles as eggs (six clutches) from two roadside ponds in central Saskatchewan the morning after they were laid. We moved each clutch into a shaded outdoor pool (42 cm height, 48 cm diameter) filled with 65 L of filtered and dechlorinated water (hereafter, water) from the adjacent R. J. F. Smith Centre for Aquatic Ecology. After hatching, we split each clutch into two pools to lower tadpole densities. We conducted a 30% water change every 2 days and fed tadpoles daily with alfalfa pellets. Tadpoles were at developmental stages 25–30 (Gosner 1960) at the time of the experiments. While we did not evaluate the developmental stage of all the tadpoles, we made sure that none of them were past stage 30 (characterized by the visible emergence of limb buds).

We collected roughly 30 adult and 30 larval diving beetles (*Dytiscus* sp.) from nearby ponds using dipnets and Gee’s minnow traps. We held the adults (29–33 mm total length) individually in plastic bowls (12 cm diameter, 9 cm height, 400 mL of water), whereas larvae (second and third instar; 42–50 mm total length) were held individually in 0.5 L plastic cups (~8 cm diameter, 12 cm height, 200 mL of water). We conducted a full water change every other day and fed both larvae and adults with a live amphipod (*Gammarus* spp.) each day.

We used 3 adult and 30 larval salamanders from a university stock colony. We housed the adults (10–12 cm total length) individually in plastic pails (~20 × 20 × 20 cm; 3 L of water), and we housed larvae (4–5 cm total length, stages 14–15 of development: [Watson and Russell 2000]) individually in plastic bowls (12 cm diameter, 9 cm height, 300 mL of water). We fed the adults and larvae with earthworms and bloodworms, respectively, every other day, followed by a full water change.

### Cue collection

#### Predator odors

Due to the large size discrepancy across individuals from life stages, we adjusted the volume of water used to collect the predator odors to standardize the cue concentration. For beetles, for which adults were roughly twice the mass of larvae, cup volume was lowered (cue concentrations for adult: 1 in 400 mL cups; larvae: 1 in 200 mL cups). Likewise, the cue concentration of salamander adults was 1 in 3 L tanks, while that for larvae was 1 in 300 mL cups, since adults were roughly 10 times the mass of larvae. To minimize diet cues in the predator odor, we first starved predators for either 24 h (juveniles) or 48 h (adults) prior to collection (Ferrari et al. 2012; Scherer and Smee 2016). We used different starvation periods for adult and juvenile predators to reflect the differences in sustaining starvation between the two life stages and avoid any starvation-related mortality. Then, we did a full water change and left the predators undisturbed for 24 h, after which, we collected all the water containing predator odor from all the predators in their tanks/cups and froze the water at −20 °C until future use.

#### Alarm cues

We obtained tadpole alarm cues by euthanizing individuals (108 total) with a blow to the head followed by rapid pulverization with a mortar and pestle (Chivers et al. 2015; Achtymichuk et al. 2022). We then diluted the product with water to reach a final concentration of 3 tadpoles per 10 mL of water, which is known to elicit a significant antipredator response in wood frog tadpoles held in 2 L of water (Chivers et al. 2015). We filtered the solution through a mesh (0.5 mm) to remove any large particles that remained. These cues were prepared fresh at the time of conditioning.

### Experimental overview

**Figure 1:**
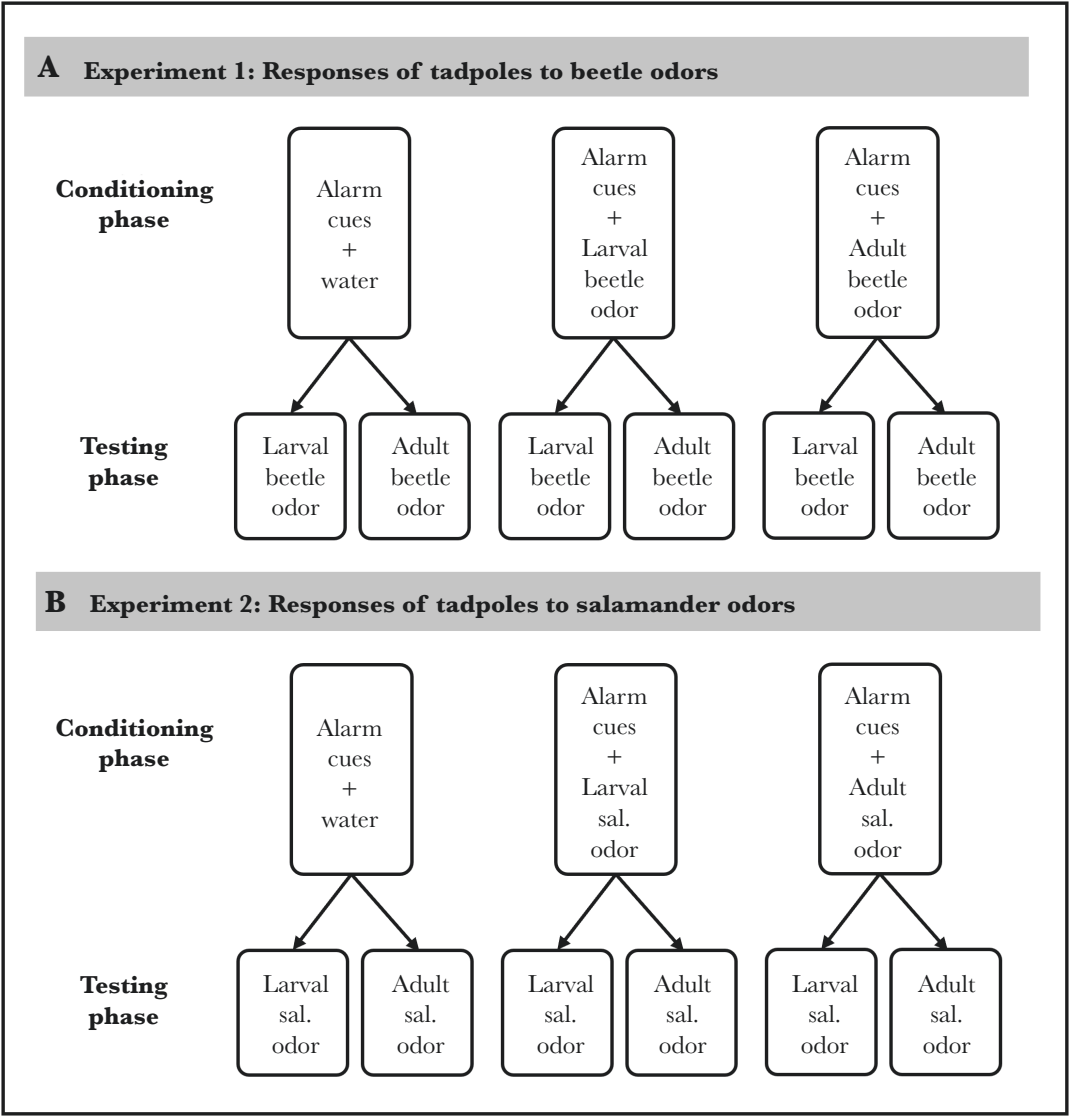
Depiction of experimental design for (A) experiment 1 and (B) experiment 2, where tadpoles were conditioned with alarm cues and either water, larval predator odor, or adult predator odor. Testing occurred 24 h later with either larval or adult predator odor. Sal. denotes salamander.

Experiment 1 and 2 followed the same experimental design (Figure 1).

#### Conditioning phase

For each experiment, we used a split-clutch design, where we arbitrarily selected 60 tadpoles from each clutch and split them into three groups of 20. Each group was then placed into a conditioning pail (~20 × 20 × 20 cm) filled with 4 L of water. We repeated this process for all 6 clutches, for a total of 18 conditioning pails (6 for each conditioning treatment). We then left the tadpoles overnight to acclimate to the pails before conditioning. The next morning, we gently lowered the water level in each pail to 2 L to reduce the amount of alarm cues that were required for an effective conditioning. The pails from each clutch were randomly assigned to one of the three conditioning treatments: (1) 10 mL of alarm cues paired with 20 mL of larval predator odor, (2) 10 mL of alarm cues paired with 20 mL of adult predator odor, or (3) 10 mL of alarm cues paired with 20 mL of water (control treatment). For each exposure, we gently injected the cues around the surface of the water using a syringe. After 1 h of the conditioning treatment, we conducted a 90% water change, which allowed us to drop the cue concentration to a very low level without having to physically handle and stress the tadpoles (Ferrari and Chivers 2009b).

#### Testing phase

On the day following conditioning, we moved individual tadpoles into 0.5-L plastic cups filled with clean water. We placed each cup in a testing tray (68 × 40 × 17.5 cm) containing 20 cups total, surrounded by 2-L of water to buffer ambient temperature fluctuations. After 1 h of acclimation, we conducted a 4-min pre-stimulus observation, recording the baseline activity level of each tadpole. We measured the activity by counting the number of times the tadpoles swam across the medial line of the cup. Following the pre-stimulus observation, we gently injected 5 ml of odor from either the adult or larval predator onto the side of the cup to minimize disturbance, and then the behavior of the tadpole was recorded during a 4-min post-stimulus observation. A reduction in line crosses is a typical antipredator response of wood frog tadpoles (Ferrari and Chivers 2009a; Fraker 2010). As in previous studies (Rivera-Hernández et al. 2022; Crane et al. 2023), only tadpoles that were actively moving during the pre-stimulus observation (defined as crossing ≥ 6 lines) were tested for reduced activity in response to the cues. The observations were conducted blind to the treatments. In total, we tested 360 tadpoles in Experiment 1 (beetle odors) and 357 in Experiment 2 (salamander odors).

### Statistical analysis

For each experiment, we first analyzed the baseline pre-stimulus line crosses using a general linear mixed model (GLMM) with the conditioning treatment (larval, adult, or sham), the test cue (larval or adult odor), and their interaction as fixed factors, and the clutch and conditioning pail as random factors (pail nested within conditioning treatment; Type I SS model). We used a Gaussian model and confirmed that the data met the model assumptions by inspecting residual plots. After confirming that the baseline data did not differ significantly across treatments in both experiments (all *P*’s > 0.05), we calculated the change in lines crossed for each trial (post–pre). We then analyzed the change in lines crossed for each experiment using a GLMM that included the conditioning treatment, the test cue, and their interaction as fixed factors, with clutch and pail (nested) as random factors (Type I SS model). For a significant main effect, in the absence of an interaction, we compared differences between test cues within each conditioning treatment with GLMMs, keeping the nested structure of pail as a random factor. In the case of a significant interaction, we split the data by the conditioning treatment and used separate GLMMs to compare the responses toward the test cues, again keeping pail as a random factor. We conducted the analyses in SPSS 26.0 with α = 0.05.

## RESULTS

### Experiment 1: Responses of tadpoles to beetle odors

**Figure 2:**
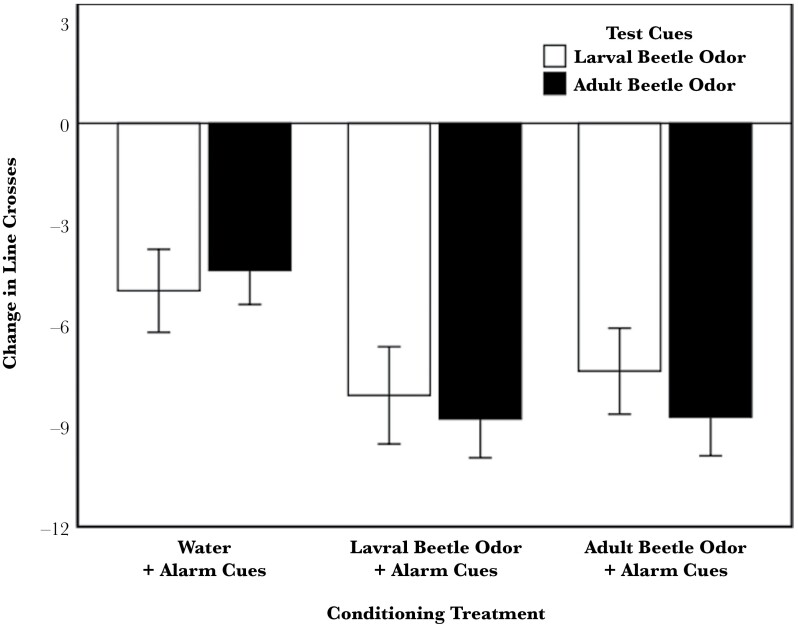
Mean (±SE) change (post–pre) in line crosses by tadpoles conditioned with alarm cues paired with either water (a sham conditioning), larval beetle odor, or adult beetle odor and then tested for a response to either the larval or adult odor.

Tadpole behavior was significantly affected by the conditioning treatment (*F*_2, 8.95_ = 4.7, *P* = 0.04), where individuals conditioned with both odors responded with antipredator behavior (reduced activity) to both testing odors in comparison to the sham conditioning treatment (water vs. adult: *F*_1, 9.93_ = 7.38, *P* = 0.02; water vs. larvae: *F*_1, 9.91_ = 7.61, *P* = 0.02; Figure 2). There was no effect of the testing cue (*F*_1, 285_ = 0.18, *P* = 0.7), clutch (*F*_5, 9.25_ = 2.29, *P* = 0.13), pail (*F*_12, 285_ = 1.18, *P* = 0.3), or an interaction between the conditioning treatment and testing cue (*F*_2, 285_ = 0.28, *P* = 0.8). Therefore, tadpoles conditioned to the odor of one beetle life stage learned to respond to that odor as well as the odor from the other life stage.

### Experiment 2: Responses of tadpoles to salamander odors

**Figure 3:**
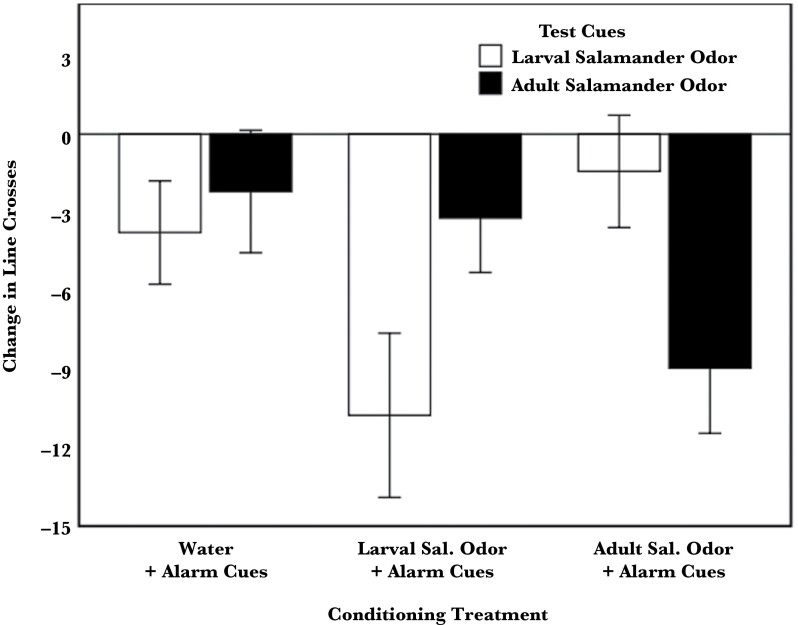
Mean (±SE) change (post–pre) in line crosses by tadpoles conditioned with alarm cues paired with either water (a sham conditioning), larval salamander odor, or adult salamander odor and then tested for a response to either larval or adult odor. Sal. denotes salamander.

The responses of tadpoles were affected by a significant interaction between the conditioning treatment and testing cues (*F*_2, 259_ = 5.74, *P* = 0.004), where individuals conditioned with the larval odor showed an antipredator response only to the larval odor (*F*_1, 87_ = 5.22, *P* = 0.025), whereas tadpoles conditioned with the adult odor responded only to the adult odor (*F*_1, 83_ = 5.20, *P *= 0.025, Figure 3). Tadpoles conditioned with water did not respond to either test cue (*F*_1, 89_ = 0.14, *P* = 0.71, Figure 3), and again, activity was not significantly affected by clutch (*F*_5, 9.69_ = 0.84, *P* = 0.55) or pail (*F*_10, 259_ = 0.83, *P* = 0.6). When tested with adult salamander odor, there was no significant difference in antipredator response between tadpoles conditioned with larval salamander odor and tadpoles conditioned with water (*t*_91_ = −0.32, *P* = 0.75). Similarly, when tested with larval salamander odor, there was no difference in antipredator response between tadpoles conditioned with adult salamander odor and tadpoles conditioned with water (*t*_105_ = 0.19, *P* = 0.85). Therefore, tadpoles conditioned to the odor of one salamander life stage did learn to respond to that odor but did not respond to the odor from the other life stage.

## DISCUSSION

Our results indicate that prey can generalize predator recognition through predator metamorphosis for some predator species but not all. Specifically, wood frog tadpoles learned to recognize diving beetles regardless of their ontogenetic stage after being conditioned to recognize one stage or the other, suggesting that the beetle odors remained consistent across metamorphosis and were perceived similarly by tadpoles. In contrast, tadpoles conditioned with either larval or adult salamanders learned to respond only to that particular life stage (i.e., they did not generalize to the other ontogenetic stage). This reveals that the pre- and post-metamorphic odors of salamanders were perceived differently, and thus from the prey’s perspective, the identity of the predator appears to be reset upon metamorphosis. Such an “identity reset” would be surprising given the wealth of literature on the ability of naïve prey to “guestimate” the risk level of novel predators that belong to the same taxonomic family as known predators (Ferrari et al. 2007b, 2008a, 2016; Davis et al. 2012).

A potential explanation for this apparent dissimilarity in the cues produced by larval and adult salamanders can be attributed to the ecological changes associated with their life history transition (Mateo 2006; Ichinose and Lenoir 2009). While adult and larval tiger salamanders may share more similarities in morphology than larval and adult diving beetles, the salamanders do undergo significant morphological and physiological changes, in addition to changes in habitat (Hahn 1962; Galey et al. 1987). As they transition from an aquatic environment to a new terrestrial environment after metamorphosis, tiger salamanders must restructure their skin to retain water and survive (Frolich and Schmid 1991). This process decreases water permeability in adult tiger salamanders by 12-fold compared to larval salamanders. After metamorphosis, densely crossed fibers in the dermis are replaced by randomly oriented loose fibers (Frolich and Schmid 1991). Single-celled dermal glands form at metamorphosis for most salamander species including Ambystomatid species like the tiger salamander (Formanowicz and Brodie 1982; Frolich and Schmid 1991). These glands contain noxious substances that can be secreted when threatened by predators, thus making them unpalatable (Brodie 1977; Formanowicz and Brodie 1982). Like most amphibians, larval tiger salamanders also switch from an ammoniotelic mode of nitrogen excretion to a ureotelic mode (Stiffler et al. 1980). Although our experiments did not account for nitrogenous waste, it is reasonable to hypothesize that the odor had different concentrations of urea and ammonia between larval and adult salamanders, which has the potential to further contribute to differences in odor profiles between the two ontogenetic stages. Thus, the combined changes in skin structure, physiology, and nitrogen excretion could be responsible for differences in odor profiles between ontogenetic stages of tiger salamanders, resulting in tadpoles’ failure to generalize odors across stages.

Compared to tiger salamanders, dytiscid beetles exhibit a more drastic change in external morphology with metamorphosis. One physiological change that could potentially affect their odor occurs in the rectal ampulla (Korschelt 1924; Eisner 1970). This is a defensive structure that is filled with water in the larval form, but after metamorphosis, becomes filled with chemicals thought to be hydrogen sulfide and/or ammonia (Korschelt 1924). When adults are stressed, they discharge these contents. During odor collection, we kept handling stress to a minimum, which may have prevented such discharge. As a result, the odor signature may not have been different enough to prevent the generalization of beetles across life stages.

Naïve prey may face a unique challenge if the cues of their predators are perceived as novel following predator metamorphosis. Hence, the ability to generalize antipredator responses to different ontogenetic stages of predators will have important fitness consequences for prey. While this study focused on predator chemical cues, predator visual cues (Chivers et al. 2013) and perhaps other kinds of cues (Seigel et al. 2021) may also be used for predator generalization. Further study can help elucidate the role of cross-sensory integration in the generalization of predator recognition and the extent of a potential “identity reset” for some metamorphosizing predators, giving them a clean slate in the eyes or nose of their prey.

## Supplementary Material

arae014_suppl_Supplementary_Data

## Data Availability

Analyses reported in this article can be reproduced using the data provided by Thapa et al. (2024).
